# The effect of using desktop VR to practice preoperative handovers with the ISBAR approach: a randomized controlled trial

**DOI:** 10.1186/s12909-023-04966-y

**Published:** 2023-12-20

**Authors:** Eva Mari Andreasen, Helen Berg, Aslak Steinsbekk, Rune Høigaard, Kristin Haraldstad

**Affiliations:** 1 Department of Health and Nursing Sciences, University of Agder, P.O. Box 422, 4604 Kristiansand, Norway; 2 Department of Health Sciences, Norwegian University of Science and Technology, P.O. Box 1517, 6025 Ålesund, Norway; 3 Department of Public Health and Nursing, Norwegian University of Science and Technology, P.O. Box 8900, 7491 Trondheim, Norway; 4 Department of Sport Science and Physical Education, University of Agder, P.O. Box 422, 4604 Kristiansand, Norway

**Keywords:** Desktop virtual reality, ISBAR approach, Nursing students, Preoperative handover, Self-practice, Structured communication, Traditional paper-based

## Abstract

**Aim:**

The aim was to investigate whether second-year undergraduate nursing students practicing the Identification-Situation-Background-Assessment-Recommendation (ISBAR) communication approach in a desktop virtual reality (VR) application had a non-inferior learning outcome compared with the traditional paper-based method when sorting patient information correctly based on the ISBAR structure.

**Methods:**

A non-inferior parallel group assessor blinded randomized controlled trial, conducted in simulation sessions as part of preparation for clinical placements in March and April 2022. After a 20-minute introductory session, the participants were randomized to self-practice the ISBAR approach for 45 minutes in groups of three in either an interactive desktop VR application (intervention) or traditional paper-based (TP) simulation. The primary outcome concerned the proportion of nursing students who sorted all 11 statements of patient information in the correct ISBAR order within a time limit of 5 min. The predefined, one-sided, non-inferiority limit was 13 percentage points in favor of traditional paper-based simulation.

**Results:**

Of 210 eligible students, 175 (83%) participated and were allocated randomly to the VR (*N* = 87) or TP (*N* = 88) group. Practicing in the desktop VR application (36% of everything correct) was non-inferior to the traditional paper-based method (22% everything correct), with a difference of 14.2 percentage points (95% CI 0.7 to 27.1) in favor of VR. The VR group repeated the simulation 0.6 times more (95% CI 0.5 to 0.7). Twenty percent more (95% CI 6.9 to 31.6) of the students in the VR group reported liked how they practiced. All the other outcomes including the System Usability Scale indicated non-inferiority or were in favor of VR.

**Conclusions:**

Self-practicing with the ISBAR approach in desktop VR was non-inferior to the traditional paper-based method and gave a superior learning outcome.

**Trial registration number:**

ISRCTN62680352 registered 30/05/2023.

**Supplementary Information:**

The online version contains supplementary material available at 10.1186/s12909-023-04966-y.

## Background

Handover of patients from one healthcare professional or organization to another is a situation in which patient safety can be threatened [1]. Handovers require sharing patient information, coordinating care, and transferring accountability and authority to the next team [2]. Structured handovers reduce patient complications, medication errors, and adverse patient events [3], whereas poor handover skills are related to misunderstandings between healthcare providers and can lead to severe consequences for patient safety [2].

When a patient undergoes surgery, a structured handover is an essential skill for healthcare workers [4–6]. Although electronic surgical checklists and digital tools to support preoperative handovers are implemented increasingly [7], previous research has demonstrated that these tools do not always improve communication and collaboration [8]. Utilization of the Identification-Situation-Background-Assessment-Recommendation (ISBAR) approach has been recognized internationally and widely adopted as a handover tool to enhance patient safety [9, 10]. ISBAR is used in clinical practice [7] and has been implemented in training and education [11].

Within nursing education lie challenges related to resources, e.g., time, instructors, and available simulation locations to practice skills, such as the ISBAR approach [12]. Furthermore, during student ward practice, there is insufficient time at clinical sites due to a decrease in number and length of hospitalization of surgery patients [13]. To help overcome some of these challenges in the educational setting, one possible solution is to use desktop virtual reality (VR) [14, 15].

VR utilizes 3D computer technology to construct an interactive virtual world, allowing users to engage with a simulated environment [16]. The level of immersion experienced by users in a virtual world may differ based on the hardware and software employed. This has led to suggestions for how to best define VR applications according to the level of immersion [17]. There are also other types of applications that have been termed desktop, screen- or computer-based VR which has been classified as non-immersive compared with VR solutions that use a head-mounted display [18]. In this publication, the term desktop VR is used. Desktop VR implies that individuals use a computer’s keyboard and mouse to observe and interact with a virtual environment displayed on the computer screen [19]. In multiplayer desktop VR versions, users can interact with each other through a representation of an avatar, sound and movement on the screen [18, 20].

Desktop VR has been used in situations, such as computer-based simulation [21], practicing surgical skills [22], and in health care education [23] for enhanced learning. However, a significant literature gap exists regarding rigorous studies with a large sample size to investigate the learning effect of using VR in nursing education [24, 25]. One study have been identified, which explored the potential benefits of nurses using desktop VR to learn handover [26]. This was a randomized controlled trial that found non-inferiority in communication performance using desktop VR for training when compared with live simulations. No studies have been found on desktop VR’s effect with learning the ISBAR approach in a preoperative handover situation with undergraduate nursing students [27].

Therefore, the aim was to investigate whether second-year nursing students self-practicing the ISBAR approach during handovers in a preoperative setting in a desktop VR application experienced a non-inferior learning outcome compared with self-practicing the traditional paper-based (TP) method to sort patient information.

## Method

### Study design

A non-inferior, parallel group assessor blinded randomized controlled trial (RCT) was conducted at three education sites. The non-inferior approach was chosen because desktop VR simulation is done virtual and thus may have some disadvantages compared with real-life skill practice [23, 28]. The study took place in March and April 2022, and was approved by the Education sector’s Service Provider (SIKT, Reference No. 305866) and the head of the pertinent study programs. No changes were made to protocols after the study commenced. The study was registered 30/05/2023 with trial number ISRCTN62680352 in the ISRCTN registry [29].

### Setting

The study was conducted as part of simulation sessions that prepared second-year undergraduate nursing students for clinical placement in medical-surgical settings. It took place in nursing programs at a university in Southern Norway (two sites) and at a university in Western Norway (one site). At the fall semester in 2020, there were 175, 153 and 145 students enrolled at the three sites, respectively. However, about half of these students were eligible, as only those undergoing clinical placements at somatic hospitals during that period could be included, in accordance with the curriculum and learning outcomes.

At all the universities, the students had been taught preoperative nursing care for surgical patients, communication between health care providers, and the ISBAR approach before the research study was launched.

The simulation set-up at each site comprised one lecture room with 12 computers with headsets for virtual desktop simulation and a room for paper-based simulation (one large room or smaller group rooms). Four instructors were used to facilitate the sessions and collect data for the study.

### Usability and pilot study

A usability study of the desktop VR application, used in the intervention in this study describes details regarding the development of the intervention [30]. In short, nine second-year undergraduate nursing students participated in the study and found the application usable overall, giving it an excellent usability score. Some technological and comprehension issues were identified, and a revised version was used in the present study.

A pilot study was conducted in February 2022 with 15 third-year undergraduate nursing students at two of the sites to try out the planned RCT activities. The pilot study’s results indicated that the planned RCT activities worked well, but it was found that the primary outcome’s difficulty level was too low. It was estimated that 20% of the participants in both groups would get everything correct on the primary outcomes [31, 32], which were used as the basis for the sample size calculation. However, in the pilot study, 80% of participants scored correctly on the primary outcome. The difficulty level was increased, and a revised test was piloted on five nursing educators, two nurses and two third-year undergraduate nursing students, all with moderate knowledge of ISBAR. In the revised test, 20% of the participants scored everything correctly, and this difficulty level was used for the present study.

### Participants

The inclusion criteria were second-year undergraduate nursing students enrolled in the nursing study program at the participating universities who had no or limited experience in supervised clinical practice in somatic hospitals. Third-year undergraduate nursing students with substantial experience in supervised clinical practice, indicating a level of competence already surpassing the specific learning outcomes targeted in this intervention, were excluded.

### Recruitment

General information about the simulation session, including that the students would be asked about participating in this study, was presented verbally during a lecture and presented in the digital learning management system for the study program. Specific information about time and place, in addition to repetition of general information, was provided in the study program schedule (at two of the sites) or sent by email (at one of the sites).

Information about the study, including voluntary study participation, was repeated at the start of the simulation session. The students were told that participation allowed the researchers to collect and use their identified data from the simulation session. Consent was provided by pressing “send” on the first questionnaire.

### Randomization and allocation

Randomization had to consider practical organization in which students participated at different times in batches of nine, 12, or 15 students; therefore, separate computer-generated randomization lists were made for each batch of students using the Microsoft Excel RAND function. Using these lists, stickers with identification (ID) numbers and allocation codes were printed. The stickers were then put in separate containers for each batch.

To allocate students into the intervention and control groups, students in the same batch got a random ID sticker from the container. Depending on the site, one ID sticker was taken out of the container and given to the student upon entering the lecture room (one site) or the stickers were given to the students after the students were seated in the lecture room (two sites). In the first case, the order the students came to the room could not be influenced and were random, and in the second case, the ID stickers were drawn from the container to ensure random order. The students wore the ID stickers visibly to allow for inspection and ensure that they participated according to allocation. The students were informed that they would be divided into two different groups that would self-practice using the ISBAR approach after the introduction, when the participants were followed to their simulation sites based on the allocation code on their ID stickers. The allocation on each ID sticker was checked again when students entered their designated sites. No errors were reported.

### Interventions

Both the intervention and control groups participated in a 20-minute introduction session that comprised information about the simulation’s practicalities and the possibility of participating in this study, answering a questionnaire, and watching a nine-minute video that explains the ISBAR approach [33]. The video was made for this study and included general information about the ISBAR approach and why, when, and how to use it. Pre-training was unnecessary and was not integrated into the schedule [20].

The simulation started after the introduction and lasted for 50 minutes. The students were informed that they should resolve any questions they had on their own, as it was a self-training situation. An instructor was present who was given a manual on what to do, including the main directive that they should only help students solve major technical problems and otherwise let the students arrive at solutions themselves.

During the simulation, the participants were divided into groups of three because the desktop VR application used in the study was designed for three participants. Previous studies had reported no difference in performance between groups of three, four, or five participants [34]. Furthermore, dividing participants into smaller groups helped reduce any potential periods of inactivity during the simulation.

#### Patient case

The patient case used in the simulation was the same for both groups (Table 1). The case was developed through an iterative process involving the research team and a group of seven clinicians and teachers, comprising a surgeon, anesthetist, emergency department nurse, surgery ward nurse, and university lecturers. The research team chose a preoperative setting because nurses play a critical role in giving and receiving patient information during handover before surgery [35]. It was decided to use a patient case in which the patient required acute gallbladder surgery because this is a common condition that typically involves similar procedures performed preoperatively. To involve three participant types and two handovers, it was decided to include nurses working on different shifts (night, day, or nurse anesthetist).

**Table 1:** The information about the patient case given to the students in both groups^a^

*The patient, Anna Hansen, born 230,462 with ID number 57957, went to the emergency ward during the night due to acute gallbladder inflammation. Acute surgery is planned. The patient was transferred to a gastro surgical ward. The patient must be prepared for acute surgery to remove the gallbladder in the surgical ward. The patient previously was diagnosed with high cholesterol and high blood pressure and takes medication for both. It has been decided that the patient will receive anesthesia and was assessed for ASA Classification 2. The patient has no allergies and no known infections. Current measurements have been taken, and the patient’s NEWS score is normal. The patient weighs 71 kg and is 172 cm tall (BMI = 24). The patient has a green peripheral venous cannula on the left hand (size 18 G) and fluid (Ringer 1000 ml) is in progress. Paracetamol 2 g and Oxycodone 2.5 mg previously were administered at* 6 a.m. *today. The patient has been fasting since midnight. The patient urinated before surgery. She is anxious about surgery.*	

*Abbreviations:* *ASA* American Society of Anesthesiologists, *NEWS* National Early Warning Score, *BMI* Body Mass Index

^a^Translated from Norwegian by the authors

#### Desktop VR application

The intervention group practiced using a desktop VR simulation called the *Preoperative ISBAR Desktop VR Application*, which was developed specifically for nursing students to practice the ISBAR approach during handover in an acute preoperative setting. The desktop VR application was created as part of a larger VR research project in healthcare education called *VirSam* (*Virtual Collaboration*) [36]. The details of its development are described below, in Supplement 1, and in a previous publication [30].

As the tasks involved a substantial amount of written text, including instructions and patient information, and the relatively little interactions with the virtual environment, it was chosen to use a desktop VR application. The academic content was developed by the research group in collaboration with a panel of seven healthcare professionals and educators. The technical solution was developed by the research group with the assistance of a hired programmer utilizing the *Unity* development platform. Based on experience from earlier application development, onboarding is important in self-practice applications [37]. Thus, the application was designed with integrated introductions for the use of desktop VR. Emphasis was placed on ensuring alignment between the learning outcome, learning activity, and assessment [38, 39], and that the application’s activities and available self-guidance covered learning tasks, supporting information, procedural information, and part-task practice [40]. A visualization of the application with the various activities are presented in a science talk [41]. Table 2 provides a summary of the steps that the participants went through in the application. Further details on VR feature design, including descriptions and classifications based on pedagogic and game elements, can be found in Supplementary file 1 [39, 40, 42, 43].

**Table 2:** Description of the different activities in the *Preoperative ISBAR Desktop VR Application*

Number	Activities	Content
1	Presentation of the ISBAR approach and familiarization with the application and each other	Animation with a voiceover explaining ISBAR and presenting the learning objectives, plus a brief overview of the tasks; instructions on how to use the arrow keys to look around and introduction of the players, represented as avatars with their own names.
2	Sort patient information based on the ISBAR approach	Animation with a voiceover instructing how to sort patient information based on ISBAR. Instructions on buttons for each ISBAR category to select where to sort provided patient information. Opportunity provided to delete patient information and sort again. ISBAR explanation available.
3	Discussion of experience with sorting	A screen displays the percentage of correct patient information sorted. A comparison of how the players sorted information is provided, and suggestions on correct sorting are available.
4	Presentation of the patient case and the professionals’ roles, and selection of the role to play	Animation with a voiceover presenting a patient case, involving three roles (nurse on night shift, nurse on day shift, and nurse anesthetist), and instructing on how to choose a role. When one player selects a role, it is no longer available to other players.
5	Handover role play	Animation with a voiceover instructing how to complete the handover. Players give and receive patient information using ISBAR sequentially. A list of all patient information and a phone are visible for the player providing patient information during the handover, and this player is instructed to mark the patient information to present first. The phone and a handover checklist are visible to the receiver of the handover. The active role player’s screen is visible to the third player not taking part in the specific handover. Explanations of ISBAR and the role playing are available.
7	Debriefing 1 – general	Animation with a voiceover describing what to do during the debriefing session. Text stating that they should discuss how each participant experienced performing the tasks in general and that they will discuss each handover in detail afterward.
8	Debriefing 2 – each player	Animation with a voiceover with instructions on how to debrief what each participant chose to highlight and say first during the handover. A screen displays a list of all patient information, highlighting the patient information that the participant marked as the information to present first. Suggested bullet points on what to discuss during the debriefing are visible. An ISBAR explanation is available.
9	Encouragement to play again	Animation with a voiceover encourages the player to practice again. A screen provides two options: practice again or end the session.

#### Traditional paper-based group

The participants in the traditional paper-based group met in-person and were placed around a table in groups of three. Due to uneven numbers, two groups comprised four students. They were given printed papers with the same explanation and tasks––including an explanation of the ISBAR approach and a list of suggestions for correct sorting (Supplementary file 2)––as the VR group (Table 2, Supplementary file 1).

#### Differences between the groups

The main difference between the groups was that the desktop VR group practiced in a virtual environment. Furthermore, in VR, the participants were represented by avatars, with their names displayed above the avatars’ heads, and instructions were delivered through animations featuring voiceovers and pop-up windows. Feedback was provided, allowing for comparing results and suggestions for correct sorting. Furthermore, feedback was also given by highlighting the first statement in each player’s handover and through debriefing sessions. Another mechanism unique to desktop VR practice was the automatic guidance between activities, with an allocated time limit, indicating progress through the practice sessions. In the VR solution, repetition was promoted through time limits, and by encouraging them to practice again after the session ended by providing a click button to start over.

### Data collection

At the beginning of the introduction, the participants completed a baseline characteristics questionnaire online. The outcome data were collected immediately after the simulation training through an online questionnaire and a written test, both with a time limit of 5 min. The ISBAR categories were not visible, i.e., the students had to remember the order and meaning.

During the data collection process, one staff member was present to provide instructions to the participants. They did not interact with the students during the data collection process and were instructed only to answer “do as you think best” in response to any questions from the students.

### Outcomes

#### Written test and scoring rules

The written test (Supplementary file 3) was used for the primary outcome and some of the secondary outcomes, as described below. All the outcomes based on the written test were scored independently by the first author and a research assistant. The assessors were presented with the set of paper responses arranged randomly in the order of submission, and the scorers were blinded to the group allocation. They both provided the same score on 95% of the participants. For the remaining 5%, two members of the research group, who also were blinded, scored and discussed the results together with the first author until a consensus was reached.

The primary and some of the secondary outcomes concerned sorting patient information within correct ISBAR categories. A score of “Everything correct” was assigned if the patient information was sorted into the correct ISBAR category, independent of the order of the patient information within the category. Furthermore, some of the patient information could be sorted correctly within two of the ISBAR categories (S and A).

#### Participant characteristics

Participant characteristics included sex, age, mother tongue (Norwegian or other), previous experience working in health care, previous experience working in a surgical ward, previous experience practicing using the ISBAR approach, and previous experience playing multiplayer PC games.

#### Implementation of the intervention

Technical and other problems were registered by asking the instructors who were present if any such issues were experienced.

#### Primary outcome

The primary outcome was the proportion of nursing students who sorted all 11 statements of patient information into the correct ISBAR order within a time limit of five minutes on the written test (Supplementary file 3). The statements with patient information were presented in random order, numbered and provided on paper. The students were instructed to “write the number on the patient information in the correct order and write the letter where the information belongs”. This outcome variable was based on earlier research [31, 32] and was tested during the pilot study.

#### Secondary outcomes

- The proportion that placed the correct patient information within each of the ISBAR categories: This outcome reports the results for each ISBAR category and provides additional information on the primary outcome by identifying the category that was best understood, as determined by the highest proportion of correct patient information placements. The outcome variable was based on prior research [31, 32] and tested during the pilot study.
- The proportion that arranged the ISBAR words correctly: This outcome came from the online questionnaire. The students were presented with the five words that comprise ISBAR, sorted in the following order “Recommendation-Background-Identification-Situation-Assessment.” They were instructed; “Sort in correct order.” A similar outcome was used in earlier research [31, 32] and tested during the pilot study.
- The proportion that sorted five statements of patient information (one for each ISBAR category) correctly based on ISBAR: This outcome was from the online questionnaire. The students were presented with the patient information sorted in the following order: “AIRBS” and asked to “sort the patient information correctly based on what you have learned today.” This outcome was made for this study and tested during the pilot study.
- Students’ experiences with the self-perceived learning outcome on five questions: This outcome came from the online questionnaire:” To which degree did you think: 1. the video about ISBAR gave you enough knowledge before you started to practice; 2. you had enough time to practice; 3. the practice method was likable; 4. the teaching activity (introduction and practice) were a good way to learn the ISBAR approach; and 5. you are confident in conducting communication in the ISBAR approach.” Five answer options were provided: 1 (*completely disagree*); 2 (*disagree*); 3 (*neither disagree/agree*); 4 (*agree*); or 5 (*completely agree*). The proportion answering agree/completely agree is reported. These outcomes were used in earlier research [31, 32] and tested during the pilot study.
- The proportion of complete runs of the practice: This outcome came from the online questionnaire. The students were asked to type the number of complete runs of the practice. A similar outcome was used in earlier research [31, 32] and tested during the pilot study.
- The simulation method’s perceived usability: This outcome came from the online questionnaire and was measured using the System Usability Scale (SUS) [44]. The SUS has 10 open-ended items, with five answer options ranging from 1 (*strongly disagree*) to 5 (*strongly agree*). The score was created by adding up responses and converting it to a 0 to 100 scale, which can be translated into a curved grading scale from A-F [45]. The SUS was viewed as a reliable test of educational technology usability [46], and the validated Norwegian version was used [47].

### Sample size calculation

A non-inferior limit of 13 percentage points was chosen for the sample size calculation based on other studies on clinical observation [31, 32, 48, 49]. Using this limit, a power (beta) of 80%, and a significance level (alpha) of 0.05, the sample size calculation demonstrated that 118 participants were needed in each group (Sealed Envelope Ltd., 2012), totaling 236 participants. For practical reasons, the maximum number of students available was 210.

### Analysis

The participant characteristics are presented descriptively. Independent sample proportion tests were used for categorical data, and independent samples t-tests were used for continuous data. The absolute difference is presented. The one-sided *p*-value with confidence intervals (CI) s on the primary outcome for non-inferiority is reported. Non-inferiority was declared if the lower limit of the one-sided 95% CI in absolute difference on the primary outcome in the VR group did not exceed 13% in favor of the control group. To present the analysis in the conventional manner, the results from a two-sided test with CIs are reported. Because none of the outcomes had more than two missing responses, all available data were used in the analyses. All analyses were performed using IBM Statistical Package for the Social Sciences (SPSS) version 28.0.0 (IBM Corp).

## Results

### Recruitment and baseline characteristics

**Fig. 1:**
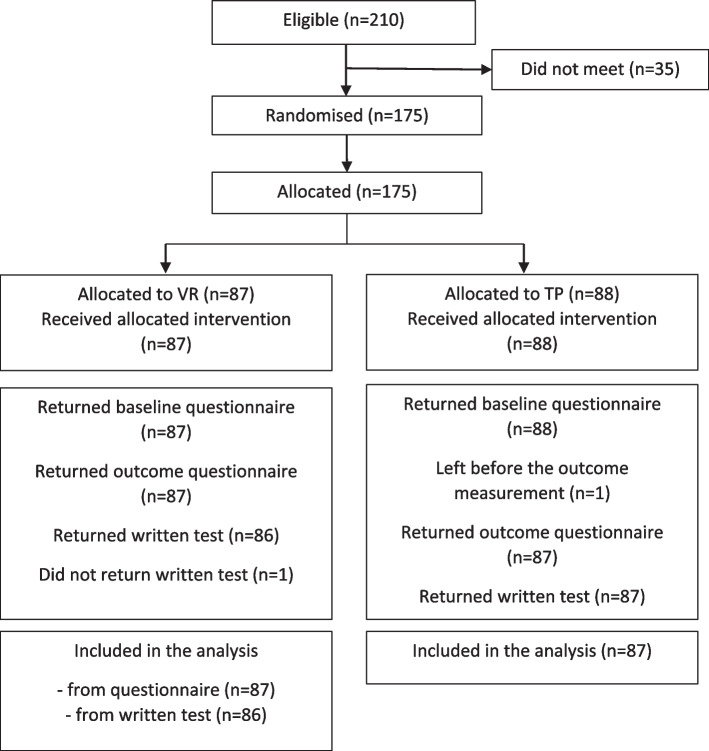
The flow of participants. Abbreviations: *VR* = desktop virtual reality; *TP* = traditional paper-based simulation

Altogether, 210 (78, 68, and 64 from each site) second-year undergraduate nursing students were eligible to participate in the study (Fig. 1). No exclusions were made, as only second-year undergraduate students attended. Ultimately, 35 did not show up for the study, so 175 participants were randomized: 87 to a desktop VR simulation group and 88 to a traditional paper-based (TP) group. One student left before the written test in the control group, and one did not return the written test in the intervention group.

The participants’ characteristics are presented in Table 3. The sample included 142 females (81.1%), and most participants were 20–24 years old. Nearly all had previously been taught the ISBAR approach, 82% reported having practiced the ISBAR approach, and 43% reported having played multiplayer PC games.

**Table 3:** Participant characteristics

Participant characteristics	All (*N* = 175)	VR group *N* = 87	TP group (*N* = 88)
	N (%)	N (%)	N (%)
Sex			
-Male	32 (18.3)	17 (19.5)	15 (17.0)
-Female	142 (81.1)	70 (80.5)	72 (81.8)
-Other	1 (0.6)		1 (1.1)
Age			
-20–24 years	122 (69.7)	63 (72.4)	59 (67)
-25–29 years	29 (16.6)	15 (17.2)	14 (15.9)
-30 years or older	24 (13.7)	9 (10.3)	15 (17)
Mother tongue			
-Norwegian	157 (89.7)	80 (92.0)	77 (87.5)
-Other	18 (10.3)	7 (8.0)	11 (12.5)
Have you previously (number answering yes):			
-Worked in healthcare?	164 (93.7)	79 (90.8)	85 (96.6)
-Worked in a surgical ward?	25 (14.3)	13 (14.9)	12 (13.6)
-Been taught the ISBAR approach?	167 (95.4)	85 (97.7)	82 (93.2)
-Practiced using the ISBAR approach?	143 (81.7)	72 (82.8)	71 (80.7)
-Played multiplayer PC-games?	76 (43.4)	45 (51.7)	31 (35.2)

Abbreviations: *VR* desktop virtual reality, *TP* traditional paper-based simulation

The groups’ characteristics were similar, but those in the VR group were somewhat younger, and a larger proportion had played multiplayer PC games earlier (Table 3).

### Implementation of intervention

The implementation of both groups was executed without major technical or practical problems. The desktop VR program had to be restarted for two of the 29 desktop VR groups because the participants could not talk to each other.

### Outcomes

**Fig. 2:**
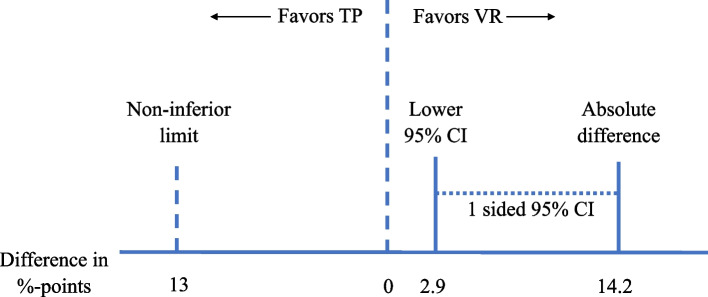
The difference between the VR and TP groups on sorting patient information, based on ISBAR. Legends: If the horizontal one-sided 95% confidence interval (CI) had crossed or been to the left of the vertical non-inferior limit, desktop virtual reality (VR) would not be non-inferior. Abbreviations: VR = desktop virtual reality; *TP* = traditional paper-based simulation

For the primary outcome, the group self-practicing on the desktop VR application (36% had everything correct) was non-inferior to the traditional paper-based group (22% had everything correct), with a difference of 14.2% points (one-sided 95% CI 2.9 to 14.2) on the primary outcome (Fig. 2, Table 4). Furthermore, the desktop VR application was superior to the traditional paper-based simulation in providing a better learning outcome (difference 14.2% points, two-sided 95% CI 0.7 to 27.1) (Table 4).

**Table 4:** Primary outcome and secondary outcomes. Numbers (%) of participants for each group and difference in percentage points with a two-sided 95% confidence interval (95% CI) between the groups

Outcome measures: number of participants who:	VR group *N* = 86	TP group *N* = 87	Difference in % points (95% CI)	*P*-value
	N (%)	N (%)		
Primary outcome: sorted 11 statements of patient information in the correct ISBAR order within a time limit of 5 minutes	31 (36.0)	19 (21.8)	14.2 (0.7 to 27.1)	0.039*
Secondary outcomes:				
Placed the correct patient information within its correct ISBAR category:				
-Identification	77 (89.5)	84 (96.6)	−7 (− 15.6 to 0.9)	0.069
-Situation	48 (55.8)	37 (42.5)	13.3 (−1.6 to 27.3)	0.081
-Background	61 (70.9)	51 (58.6)	12.3 (−1.9 to 25.8)	0.090
-Assessment	44 (51.2)	28 (32.2)	19 (4.3 to 32.6)	0.011*
-Recommendation	77 (89.5)	80 (92)	−2.4 (−11.6 to 665)	0.583
Arranged ISBAR words correctly	87 (100)	84 (97.7)	2.3 (−2.2 to 8.1)	0.153
Proportion who sorted all five pieces of patient information correctly	60 (69)	61 (70.9)	−2 (−15.4 to 11.6)	0.778

Abbreviations: *VR* desktop virtual reality, *TP* traditional paper-based simulation. *p < 0,05

For the secondary outcomes, the desktop VR groups had an average of 1.8 complete runs of the practice (distribution in Table 5), compared with 1.2 runs in the TP group (mean difference 0.6, two-sided 95% CI 0.5 to 0.7, *P*-value < 0.001).

**Table 5:** The number of completed runs (briefing-rehearsal-debriefing)

Number of completed runs	VR group *N* = 87	TP group *N* = 86
	N (%)	N (%)
0		1 (1.1)
1	19 (21.8)	67 (76.1)
2	65 (74.7)	16 (18.2)
3	3 (3.4)	2 (2.3)

Abbreviations: *VR* desktop virtual reality, *TP* traditional paper-based

The outcomes placing the correct patient information within its correct ISBAR category were similar in the two groups, except for the category *assessment* (a difference of 19 percentage points in favor of VR, two-sided 95% CI 4.3 to 32.6). The other outcomes on arranging the ISBAR words and pieces of patient information correctly were similar in the two groups.

The outcomes from the students’ experiences with the self-perceived learning outcome indicated that the desktop VR group performed either non-inferior or better than the TP group (Table 6). The VR group participants reported that they liked this type of practice better (difference: 20% points). For the perceived usability of the simulation method, the VR group provided an SUS mean score of 78.6, which was non-inferior to the TP group, with a mean of 76.3. Both groups got a Grade C based on Bangor, Kortum [47] grading scale.

**Table 6:** Secondary outcomes on the students’ experiences with self-perceived learning outcomes and perceived usability of simulation methods. Numbers (%) of participants for each group and difference in percentage points with a two-sided 95% confidence interval (95% CI) between groups

Outcome measures	VR group *N* = 86	TP group *N* = 87	Absolute diff. in % points (95% CI)	*P*-value
	N (%)	N (%)	Mean (SD)	
Number of participants who reported (%):				
- Enough training from the ISBAR video before practicing	67 (77.7)	66 (76.7)	0.3 (−12.2 to 12.8)	0.750
- Had enough time to practice	67 (77)	66 (76.7)	0.3 (−12.2 to 12.8)	0.750
- The practice method was likable	75 (86.2)	56 (66.7)	19.5 (6.9 to 31.6)	0.003*
- Training and practice were good ways to learn the ISBAR approach	74 (86)	63 (75.9)	10.1 (−1.7 to 21.9)	0.110
- Were confident communicating with the ISBAR approach	50 (57.5)	37 (44)	13.4 (−1.5 to 27.6)	0.056
Perceived usability of the simulation method: - System Usability Scale (range 0–100, higher better) mean score (standard deviation SD)	Mean 78.6 (SD 14.2)	Mean 76.3 (SD 18.4)**	Mean diff. 2.3 (−1.8 to 6.4)	0.272

Abbreviations: *VR* desktop virtual reality, *TP* traditional Paper-based. *p < 0,05. **N = 84

## Discussion

There was a superior learning outcome of the *Preoperative ISBAR Desktop VR Application* on sorting patient information correctly based on the ISBAR approach used for handovers in a preoperative setting, compared to traditional paper-based simulation. Most of the other outcomes indicated that desktop VR was non-inferior, but those practicing with desktop VR liked the practice better and practiced more.

### More likeable, yet better learning outcome

It was somewhat surprising that desktop VR was found to be superior to traditional practice. The study was designed as a non-inferior study, as VR can offer some disadvantages due to technical and comprehension issues [30, 50], along with a lack of face-to-face communication when practicing in desktop VR [51]. Furthermore, one review of randomized controlled trials investigating desktop virtual simulation compared with traditional learning found no clear differences when measuring learning outcomes [15], and another review found that virtual simulation provided a non-inferior outcome on teamwork attitudes when learning interprofessional team communication [26]. This study’s findings were not in line with expectations and the review’s findings. Thus, more studies that elicit a superior outcome from desktop VR are required before the review findings’ conclusion can be challenged.

Although desktop VR has the same learning outcome as traditional simulation, in this study and others [23, 52], participants reported VR as being more likable. However, even if this study found that the participants’ preferred simulation method (desktop VR) resulted in a better learning outcome, this does not seem to be the general rule. Previous systematic reviews on e-learning that investigated objective learning outcomes and satisfaction found a negative association between these two factors [53, 54], i.e., higher satisfaction is associated with lower learning outcomes. In an RCT, it was found that students who participated in an active learning approach self-reported lower learning outcomes than those in a passive learning approach [55]. However, when objective measures of learning were assessed, students in the active learning group demonstrated higher learning outcomes than their peers in the passive learning group. This indicates that student satisfaction with learning and self-reported learning are not accurate indicators of objective learning outcomes.

### Potential mechanisms behind the findings

Aside from the possibility of a chance finding, we suggest five possible mechanisms to explain the superior effect and likability of desktop VR found in this study.

The first is automated individual feedback. A VR application, like the one in this study, can be programmed to provide instant feedback. Feedback on performance is crucial to learning and can be enhanced by timely, specific, and learner-targeted feedback [56]. Drawing on the theoretical perspective of deliberate practice, feedback can function as a stimulus to continuing practicing [57], thereby promoting learning. Several studies have found feedback to be a mechanism for learning through technological learning activities [58] and game-based learning [59–62].

The second mechanism is that in a virtual environment, players are represented by avatars, which can create a sense of anonymity that can increase enjoyment of the experience [63]. Furthermore, learners in a traditional face-to-face learning environment have reported that they may feel self-conscious about speaking up in front of others, fearing judgment or criticism [64]. Based on Chen and Kent [65], one reason can be that the anonymity provided through avatars can create a sense of security that can shield learners from feeling embarrassed or singled out when making mistakes. Another aspect is that avatars can create a more neutral learning environment by reducing the impact from physical attributes, e.g., sex [66] and ethnicity [67], to help prevent unconscious biases.

The third suggested mechanism is related to how information is provided during the simulation. The use of visual instructions as a tool for learning has been investigated in several studies, and it has been found that both visual appearance of educational content in VR [68] and displaying extra information when practicing can benefit learning [69].

The fourth mechanism is automatic guidance supporting progression during practice. Automatic guidance in VR can exert both positive and negative effects on learning, depending on the context and the type of guidance provided [70]. For example, excessive automatic guidance can lead to a phenomenon known as the “guidance paradox” [70], in which learners become overly reliant on guidance and fail to develop necessary skills and knowledge to perform tasks independently. However, the observed effect in this study indicates that the positive aspects of helping learners navigate the simulation can overcome negative aspects if automatic guidance is used optimally.

The fifth and final mechanism that we suggest is repetition. A notable finding in this study and others [71] is that those practicing in VR repeated the simulation more often during the same practice session. Repetitive simulation practice has been found to enhance learning outcomes [72, 73].

### Strengths and limitations

This study’s main strength was the randomized controlled trial design, a relatively high number of students and a blinded assessment of the primary outcome. However, although recent findings suggest that blinding is less important than previously thought [74], this study’s limitation was that it was not possible to blind the students due to the study’s nature. Furthermore, the study evaluated only one type of desktop VR application, which may limit the findings’ generalizability to other VR applications. Finally, the learning outcome was measured immediately after practice, which means that the intervention’s long-term impact was not measured.

## Conclusion

This study was designed to investigate whether nursing students, self-practicing the ISBAR approach in desktop VR, achieved a non-inferior learning outcome compared with self-practicing traditional practice, which was confirmed. However, it also was found that desktop VR provided superior learning outcomes. Furthermore, the students preferred using desktop VR and practiced more within the given time limit. This interactive desktop VR can be recommended as a practical and engaging way for second-year undergraduate nursing students to self-practice the ISBAR approach.

### Supplementary Information

**Additional file 1.** Presentation of the Preoperative ISBAR Desktop VR Application with the desktop virtual reality feature description and classification according to pedagogic- and game elements.

**Additional file 2.** ISBAR practice – sorting and role play.

**Additional file 3.** Individual final assignment and scoring rules.

## Data Availability

The datasets used during the current study available from the corresponding author on reasonable request. It is also available from the Service Provider for the Education Sector (SIKT, reference 305866) repository at https://sikt.no/veiledning-bestille-data, where the persistent web link can also be found.

## Abbreviations

ASA: American Society of Anesthesiologists
BMI: Body Mass Index
ISBAR: Identification, Situation, Background, Assessment, and Recommendation
NEWS: National Early Warning Score
SUS: System Usability Scale
TP: Traditional paper-based
VR: Virtual reality.
