# Supplementary material for: The effect of using desktop VR to practice preoperative handovers with the ISBAR approach: a randomized controlled trial
Source: BMC Med Educ. 2023 Dec 20;23:983. doi: 10.1186/s12909-023-04966-y (PMC10731819; doi:10.1186/s12909-023-04966-y)
Supplement: Supplementary file 1 — Additional file 1. Presentation of the Preoperative ISBAR Desktop VR Application with the desktop virtual reality feature description and classification according to pedagogic- and game elements. [file 12909_2023_4966_MOESM1_ESM.docx]

**Supplementary file 1**. Presentation of the Preoperative ISBAR Desktop VR Application with the desktop virtual reality feature description and classification according to pedagogic- and game elements.

| **Number** | **Sequence** | **Content** | **Reason for feature and design** |
| --- | --- | --- | --- |
| **1** | Instruction: Name and group register | Insert name. Group allocation number with instruction to choose group | Designed to make the participant names visible to all group members throughout the learning session |
| **2** | Instruction: Introduction to ISBAR^*^ | Animation with a voiceover explaining ISBAR, presenting the learning objectives, and a brief overview of the tasks | To provide guidance, conceptualizing and planning during task completion |
| **3** | Learning task: Familiarization with desktop VR and each other | Instructions on how to use the arrow keys to look around and introduce the players, represented as avatars with their own name, to each other | To promote guidance and stimulate exploration |
| **4** | Instruction: Sort patient information | Animation with voiceover instructing how to sort patient information according to ISBAR and how to get additional information | Guidance to develop mental models and cognitive strategies to complete the learning task |
| **5** | Task: Sort patient information   Supportive information available | Buttons for each ISBAR category to select where to sort provided patient information  Opportunity to delete the patient information and to sort it again. ISBAR explanation is available | Featured learning mechanics: Part-task practice for a considerable amount of repetition  Time limit for the task to promote several runs of the complete learning session |
| **6** | Task: Discussion of experience with sorting | A screen displays the percentage of correct patient information sorted  A comparison of how the players sorted and a suggestion for the correct sorting | Information available to promote peer-student-evaluation and group discussion  Percentage visible to stimulate motivation and competition between players |
| **7** | Instruction and task:  Patient case and choose a professional role | Animation with voiceover presenting a patient case, the three roles involved (nurse on night shift, nurse on day shift, and nurse anesthetist), and how to choose a role | Guidance to develop mental models and cognitive strategies to complete the learning task |
| **8** | Instruction and task: Role description and choose a role | Description of the three roles involved with pictures symbolizing the roles  When one player selects a role, the role is no longer available for other players | Guidance to develop mental models and cognitive strategies to complete the learning task |
| **9** | Instruction: Handover role play | Animation with voiceover instructing how to complete the handover  Players give and receive patient information in their professional roles using ISBAR | Provision of procedural information to promote guidance and strategy planning |
| **10** | Instruction: Handover role play | Animation of a written summary of the next task | Guidance and demonstration for developing mental models and cognitive strategies to complete the learning task |
| **11** | Task: Handover role play | A list of all patient information and a phone is visible for the player giving patient information during handover  The phone and a handover checklist are visible to the receiver of the handover  The active role player’s screen is visible to the other players.  ISBAR explanation and explanation of the role play is available | Part-task practice for considerable amount of repetition  Time limit for the task to promote several runs of the complete learning session  Facilitation of observation, realism, and immersion |
| **12** | Instruction: Debriefing 1 | Animation with a voiceover describing what to do during the debriefing session | Guidance and demonstration for developing mental models and cognitive strategies to complete the learning task |
| **13** | Task: Debriefing 1 | Text stating that they should discuss how each participant experienced doing the tasks in general and that they will discuss each participant in detail afterward | Supportive procedural instructions for guidance |
| **14** | Instruction: Debriefing 2 | Animation with a voiceover with instructions to debrief what each participant chose to highlight and say first during the handover | Guidance and demonstration to facilitate for an optimal self-guided debrief |
| **15** | Task: Debriefing 2 | A screen displays a list of all patient information, reported with highlighting the patient information the participant has clicked as the information to present first.  Suggested bullet points on what to discuss during the debriefing are visible  ISBAR explanation available | Guided self-debrief designed for virtual simulation with procedural instructions  A learning task-cantered visualized approach of each participant’s sorting |
| **16** | Instruction: Debriefing closure | Animation with a voiceover with encouragement to practice again | Guidance and demonstration |
| **17** | Task: Final practice and ending | A screen with available options: to practice again or end the practice | To give procedural information, so it is evident for participants what to do and motivate for repetition |

*ISBAR: identification-situation-background-assessment-recommendation.
