# Supplementary material for: The effect of using desktop VR to practice preoperative handovers with the ISBAR approach: a randomized controlled trial
Source: BMC Med Educ. 2023 Dec 20;23:983. doi: 10.1186/s12909-023-04966-y (PMC10731819; doi:10.1186/s12909-023-04966-y)
Supplement: Supplementary file 2 — Additional file 2. ISBAR practice – sorting and role play. [file 12909_2023_4966_MOESM2_ESM.docx]

# **Supplementary file 2.** ISBAR practice – sorting and role play.

## **Introduce yourself to each other**

- What's your name?
- Which experiences do you have with structured communication?

(The person born earliest in the year starts the presentation)

## **ISBAR Explanation**

| **I – Identification** | Who are you, and who is the patient? |
| --- | --- |
| **S – Situation** | Why are to making contact? |
| **B – Background** | A brief and relevant medical history |
| **A – Assessment** | Important information about the patient's condition |
| **R – Recommendation** | Suggestions for what should happen next |

## **Learning task: Sort patient information**

You are a nurse on day duty at a surgical ward. The first task is to sort patient information according to the ISBAR approach. The information you have to sort is shown in the table.

Enter the letter in ISBAR by yourselves that you think the information belongs to in the table window.

You have five minutes and 30 seconds to sort. When everyone has finished sorting, compare each other's results, and discuss with the others in the group.

| **Information** | **Sorting (write a letter from ISBAR)** |
| --- | --- |
| A planned acute surgery to remove the gallbladder |  |
| The patient's name is Anna Hansen |  |
| Weight 71 kg, height 172 cm (KMI = 24) |  |
| No allergies |  |
| I consider that you continue preparations for surgery |  |
| No infection |  |
| Transferred from the emergency ward due to inflammation in the gallbladder |  |
| NEWS-score 0 |  |
| Green peripheral venous cannula on the left hand (size 18G), fluid in progress |  |
| The patient's birth number is 23062 57957 |  |
| Fasting since midnight |  |
| Medicated for high cholesterol and high blood pressure |  |
| I consider that the patient is ready for surgery |  |
| From previous high cholesterol and high blood pressure |  |
| The preoperative checklist has been updated and signed |  |
| She is anxious about the surgery |  |
| She claims to feel well today |  |
| Paracetamol 2 g and Oxycodone 2,5 mg intravenous at 6 am |  |
| «Your name» from the gastro medical ward |  |
| Fluid (Ringer) 1000 ml in progress |  |
| Complete the preoperative checklist and sign |  |
| Will receive anesthesia, ASA classification 2 |  |
| Got acute inflammation in the gallbladder |  |
| She talked to the surgeon and the anesthetist |  |
| She has peed/urinated |  |

## **Discussion**

- Go through sorting. What have you sorted alike/different?
- What could be the reason you have sorted differently?

## **Chose role**

You are nurses and are responsible for giving and receiving information about the patient. Last night, the patient Anna Hansen was transferred from the emergency to the surgical ward due to acute inflammation in the gall bladder. The patient needs immediate surgical help to remove the gall bladder. There are three roles involved in this. Agree with the others on what role each individual should have.

**Roles**

**Nurse at surgical ward – night shift**Receives patients who arrive at night, for example, transferred from the emergency ward

**Nurse at surgical ward – day shift**This role takes over responsibility for the patient from the night shift. Responsible for the last phase of patient preparation before surgery. Must pass on information to nurse anesthetists about the patient and preparations made before surgery.

**A nurse anesthetist in the operating department**Takes over responsibility for the patient from the day shift nurse when the patient arrives in the operating department. Ensures all relevant preparations are done, and all information has been received.

## **Task: Handover role play in ISBAR structure**

There are two tasks: To be the sender who gives information and to be the receiver who receives the information. A tip is only to provide a few details first. Then it should be the recipient who asks for the other information. Take the patient's case as a starting point. Practice using the ISBAR approach when you give and receive patient information from each other.

**Case:***The patient, Anna Hansen, born 230462 with ID number 57957, went to the emergency ward during the night due to acute gallbladder inflammation. Acute surgery is planned. The patient was transferred to a gastro surgical ward. The patient must be prepared for acute surgery to remove the gallbladder in the surgical ward. The patient previously was diagnosed with high cholesterol and high blood pressure and takes medication for both. It has been decided that the patient will receive anesthesia and was assessed for ASA Classification 2. The patient has no allergies and no known infections. Current measurements have been taken, and the patient's NEWS score is normal. The patient weighs 71 kg and is 172 cm tall (BMI = 24). The patient has a green peripheral venous cannula on the left hand (size 18 G) and fluid (Ringer 1000 ml) is in progress. Paracetamol 2 g and Oxycodone 2.5 mg previously were administered at* 6 a.m. *today. The patient has been fasting since midnight. The patient urinated before surgery. She is anxious about surgery.*

The preoperative checklist is updated and signed by the nurse on day duty. It is assessed whether the patient is ready for surgery. On the day shift, final preoperative preparations are completed. This could, for example, be to ensure that the patient urinates immediately before surgery and to check that the patient has not had a newly occurring respiratory infection. This information must be passed on during the handover to the nurse anesthetist.

## **Debrief**

Briefly discuss how you experienced the task.

- How did you experience doing the task?
- What would you like to get feedback on from the others in the group?
- What did you find difficult, and what did you achieve?
- What do you want to train more on?
