# Supplementary material for: The effect of using desktop VR to practice preoperative handovers with the ISBAR approach: a randomized controlled trial
Source: BMC Med Educ. 2023 Dec 20;23:983. doi: 10.1186/s12909-023-04966-y (PMC10731819; doi:10.1186/s12909-023-04966-y)
Supplement: Supplementary file 3 — Additional file 3. Individual final assignment and scoring rules. [file 12909_2023_4966_MOESM3_ESM.docx]

# **Supplementary file 3.** Individual final assignment and scoring rules.

# **Individual final assignment**

Your ID:___________________ (the number on the ID tag)

You are going to do a handover to a colleague. Below is the information you wish to pass on about the patient. Please read through all the information before you start sorting. You have 5 minutes to write down the order you learned and practiced today.

To avoid writing so much text, please only write the **number** on the patient information in the correct order and write the **letter** where the information belongs.

| Number | Write the letter |
| --- | --- |
|  |  |
|  |  |
|  |  |
|  |  |
|  |  |
|  |  |
|  |  |
|  |  |
|  |  |
|  |  |
|  |  |

**Handover information**

| **Number** | **Patient information** |
| --- | --- |
| 1 | The patient was in surgery eight hours ago |
| 2 | I suggest you contact a doctor to arrange more painkillers |
| 3 | The patient’s score on the pain scale (NRS) has now increased to 7 |
| 4 | The patient has received painkillers as prescribed |
| 5 | I am nurse NN (name) at the orthopedic ward |
| 6 | The patient has earlier been diagnosed with diabetes type 2 |
| 7 | The surgical wound looks normal |
| 8 | The patient’s name is Harald S. Plassen |
| 9 | NEWS-score er 0 |
| 10 | The patient is born in 191040 |
| 11 | The patient takes prescription medication for his diabetes type 2 |

# **Scoring rules for the individual final assignment**

Coding:  **Variable *I*:**

1. Pass = Only the numbers 5, 8 and 10
2. Pass = The letter *I* must be written corresponding to the numbers 5, 8 and 10
3. Fail = Only some of the numbers 5, 8, and 10 are documented
4. Fail = More numbers in addition to 5, 8 and 10
5. Fail = No letters are written
6. Fail also if the candidate has written arrows to change the numbers after writing them, even if the answer with arrows is correct
7. Is assessed as yes/no/empty, Yes=1, No=0, Empty=0
8. Yes is right according to the criteria above, no is wrong according to the criteria above, or blank

**Variable *S*:**

1. Pass = Only the numbers 1 and 3, or one of them
2. Pass = The letter *S* must be written corresponding to the numbers 1 and 3, or one of them
3. Fail = More numbers in addition to 1 and/or 3
4. Fail = No letters are written
5. Fail also if the candidate has written arrows to change the numbers after writing them, even if the answer with arrows is correct
6. Is assessed as yes/no/empty, Yes=1, No=0, Empty=0
7. Yes is right according to the criteria above, no is wrong according to the criteria above, or blank

**Variable *B*:**

1. Pass = Only the numbers 6 and 11
2. Pass = The letter *B* must be written corresponding to the numbers 6 and 11
3. Fail = One of the numbers 6 and 11
4. Fail = More numbers in addition to 6 and 11
5. Fail = No letters are written
6. Fail also if the candidate has written arrows to change the numbers after writing them, even if the answer with arrows is correct
7. Is assessed as yes/no/empty, Yes=1, No=0, Empty=0
8. Yes is right according to the criteria above, no is wrong according to the criteria above, or blank

**Variable *A*:**

1. Pass = Only 4, 7, 9 and 1 or 3
2. Pass = The letter *A* must be written according to the numbers 4, 7, 9, and alternatively 1 or 3
3. Fail = Some of the numbers 4, 7, or 9 are documented
4. Fail = More numbers in addition to 4, 7, 9, and alternatively 1 or 3
5. Fail = No letters are written
6. Fail also if the candidate has written arrows to change the numbers after writing them, even if the answer with arrows is correct
7. Is assessed as yes/no/empty, Yes=1, No=0, Empty=0
8. Yes is right according to the criteria above, no is wrong according to the criteria above, or blank

**Variable *R*:**

1. Pass = Only 2
2. Pass = The letter *R* must be written corresponding to the number
3. Fail = More numbers in addition to 2
4. Fail = No letter are written
5. Fail also if the candidate has written arrows to change the numbers after writing them, even if the answer with arrows is correct
6. Is assessed as yes/no/empty, Yes=1, No=0, Empty=0
7. Yes is right according to the criteria above, no is wrong according to the criteria above, or blank

**The variable number of patient information that is documented with correct value:**

1. The number of passes within all categories corresponds to the criteria for each ISBAR variable
2. Patient information passed is scored from 0-11. The score of 11 = all 11 patient information documented with the correct letter

**Variable number of patient information with the correct corresponding number in the correct order from *I* to the first error:**

1. The number of passes within all categories corresponds to the criteria for each *ISBAR* variable
2. Fail = if there is only the correct patient information in the right row, regardless of whether it is the correct corresponding letter
3. Fail = only used the patient information and put letters on
4. It is assessed as a value from 0-5. The score of 5 = The information order of 5 pieces of information (*ISBAR* is documented with correct patient information
5. The information category that may have errors shall not be counted (i.e., if the first error is in *A*, a value of 3, which will be *I-B,* is scored)

**Main outcome Everything is correct**:

1. Assessed as yes/no/empty: Yes = 1, No = 0, Empty = 0
